# Supplementary material for: Modulation of Immunity, Antioxidant Status, Performance, Blood Hematology, and Intestinal Histomorphometry in Response to Dietary Inclusion of Origanum majorana in Domestic Pigeons’ Diet
Source: Life (Basel). 2023 Feb 28;13(3):664. doi: 10.3390/life13030664 (PMC10051733; doi:10.3390/life13030664)

Test Report

Report Number
(91016)

Project Number
(8001916)

Report Number: 91016

Project Number: 8001916

Date of Issue: 9/20/2022

Customer: Hala Yehia abd El- Wahab

Address:

Sample ID: 9006573 **Date Sample(s) Received:** 8/31/2022

Customer's Reference ID: **Matrix:** Plant

Analyte / Parameter	Test Method	Description
(-)-Adrenaline	ACAL-APR-37-00	Value:0.383%<br>Retention time:36.842 min
(+-)-P-menth-1-en-4-ol	ACAL-APR-37-00	Value:11.773%<br>Retention time:11.907 min.
(([(2-Aminopropanoyl)amino]acetyl)amino) acetic acid	ACAL-APR-37-00	Value:0.097%<br>Retention time:22.696 min
(+-)-Linalool	ACAL-APR-37-00	Value:0.640%<br>Retention time:10.841 min.
(+)-Nor pseudoephedrine	ACAL-APR-37-00	Value:0.114%<br>Retention time:12.566 min.
.Alpha.-Fellandrene	ACAL-APR-37-00	Value:2.069%<br>Retention time:9.308 min.
Alpha.-Thujene	ACAL-APR-37-00	Value:0.911%<br>Retention time:7.938 min.
.Gamma.-Terpinen	ACAL-APR-37-00	Value:5.528%<br>Retention time:10.223 min.
1-(3-Ethoxyphenyl)-2-propanamine	ACAL-APR-37-00	Value:0.076%<br>Retention time:22.031 min.
1,2-Dihydro-3-methoxy-2-oxo-9(10H)-Acridinone ,	ACAL-APR-37-00	Value:0.201%<br>Retention time:24.468 min.
1,2-Dimethylpropanamine	ACAL-APR-37-00	Value:0.070%<br>Retention time:23.116 min.
10-Heptyl-10-octylicosane	ACAL-APR-37-00	Value:0.979%<br>Retention time:36.521 min.
1-Methyloctadecylamine	ACAL-APR-37-00	Value:0.060%<br>Retention time:19.618 min.
1R-.alpha.-Pinene	ACAL-APR-37-00	Value:0.173%<br>Retention time:8.078 min.
2-(2-Aminopropyl)phenol	ACAL-APR-37-00	Value:0.087%<br>Retention time:19.537 min.
2-Fluoro-5-[1-hydroxy-2-(methylamino)ethyl] phenol	ACAL-APR-37-00	Value:0.202%<br>Retention time:32.779 min.

Analytical Chemistry Unit (ACAL)
www.acal.edu.eg Tel: +20(88)2412315
info@acal.edu.eg Fax: +20 (88) 2080799
Mob: +20(101)9343637
+20(115)2854546
Assiut University
ACAL

Test Report

Report Number
(91016)

Project Number
(8001916)

Report Number: 91016
Project Number: 8001916
Date of Issue: 9/20/2022
Customer: Hala Yehia abd El- Wahab
Address:

Sample ID: 9006573 **Date Sample(s) Received:** 8/31/2022
Customer's Reference ID: **Matrix:** Plant

Analyte / Parameter	Test Method	Description
2-Isopropyl-5-methyl-phenol	ACAL-APR-37-00	Value:0.142%<br>Retention time:13.044 min.
2-Methyl-6-(4-methylphenyl) pyridine	ACAL-APR-37-00	Value:0.647%<br>Retention time:27.848 min.
2-Methylpiperazine	ACAL-APR-37-00	Value:0.0760%<br>Retention time:15.521 min.
3-(1-Pyrrolyl)Phenol	ACAL-APR-37-00	Value:1.660%<br>Retention time:29.982 min.
3-Carene	ACAL-APR-37-00	Value:1.100%<br>Retention time:12.636 min.
3-Chloro-4-methyl-2-pentanol	ACAL-APR-37-00	Value:0.544%<br>Retention time:8.369 min.
3-Hexanol	ACAL-APR-37-00	Value:0.566%<br>Retention time:8.200 min.
3-Hydroxy-N-methylphenethylamine	ACAL-APR-37-00	Value:0.102%<br>Retention time:15.667 min.
3-Methylaminopropylamine	ACAL-APR-37-00	Value:0.090%<br>Retention time:8.608 min.
4 (10)-Thujene	ACAL-APR-37-00	Value:3.937%<br>Retention time:9.745 min.
5-(2-Aminopropyl)-2-methylphenol	ACAL-APR-37-00	Value:0.060%<br>Retention time:12.898 min.
9,10-dehydro-Isolongifolene	ACAL-APR-37-00	Value:0.750%<br>Retention time:18.051 min.
9-Octylicosane	ACAL-APR-37-00	Value:3.071%<br>Retention time:44.291 min.
Alpha. Terpinene	ACAL-APR-37-00	Value:6.777%<br>Retention time:9.517 min.
Alpha. Terpineol	ACAL-APR-37-00	Value:3.566%<br>Retention time:12.030 min.
Alpha.- Terpinolen	ACAL-APR-37-00	Value:1.529%<br>Retention time:10.718 min.

Test Report

Report Number
(91016)

Project Number
(8001916)

Report Number: 91016
Project Number: 8001916
Date of Issue: 9/20/2022
Customer: Hala Yehia abd El- Wahab
Address:

Sample ID: 9006573 **Date Sample(s) Received:** 8/31/2022
Customer's Reference ID: **Matrix:** Plant

Analyte / Parameter	Test Method	Description
Alpha.,p-Dimethyl styrene	ACAL-APR-37-00	Value:0.103%<br>Retention time:11.954 min.
Beta Myrcene	ACAL-APR-37-00	Value:0.531%<br>Retention time:9.028 min.
Boric acid, ethyl-, didecyl ester	ACAL-APR-37-00	Value:2.479%<br>Retention time:40.124 min.
Caryophyllene	ACAL-APR-37-00	Value:1.492%<br>Retention time:15.037 min.
Caryophyllene oxide	ACAL-APR-37-00	Value:0.519%<br>Retention time:18.173 min.
Cathine	ACAL-APR-37-00	Value:0.023%<br>Retention time:11.546 min.
Cymol	ACAL-APR-37-00	Value:4.157%<br>Retention time:9.651 min.
Dextroamphetamine	ACAL-APR-37-00	Value:0.045%<br>Retention time:15.393 min.
dl-Alanyl-dl-.alpha.-amino-n-butyric acid	ACAL-APR-37-00	Value:0.055%<br>Retention time:21.676 min.
dl-Alanyl-dl-leucine	ACAL-APR-37-00	Value:0.063%<br>Retention time:18.814 min.
Dl-Alanyl-dl-methionine	ACAL-APR-37-00	Value:0.034%<br>Retention time:19.758 min.
Dl-Alanyl-dl-norleucine	ACAL-APR-37-00	Value:0.991%<br>Retention time:42.915 min.
Docosane	ACAL-APR-37-00	Value:17.631%<br>Retention time:42.356 min.
Heneicosane	ACAL-APR-37-00	Value:0.113%<br>Retention time:19.968 min.
Hexahydrofarnesyl acetone	ACAL-APR-37-00	Value:0.489%<br>Retention time:21.763 min.
L-Alanyl-l-alanyl-l-alanine methyl ester	ACAL-APR-37-00	Value:0.141%<br>Retention time:13.312 min.

Test Report

Report Number
(91016)

Project Number
(8001916)

Report Number: 91016

Project Number: 8001916

Date of Issue: 9/20/2022

Customer: Hala Yehia abd El -Wahab

Address:

Sample ID: 9006573 **Date Sample(s) Received:** 8/31/2022

Customer's Reference ID: **Matrix:** Plant

Analyte / Parameter	Test Method	Description
n-Eicosane	ACAL-APR-37-00	Value:0.387%<br>Retention time:33.630 min.
N-Methoxy-1-ribofuranosyl-4<br>-imidazolecarboxylic amide	ACAL-APR-37-00	Value:0.130%<br>Retention time:23.046 min.
N-Methyl-N-(4-pentenyl)amine	ACAL-APR-37-00	Value:0.0370%<br>Retention time:13.417 min.
n-Octacosane	ACAL-APR-37-00	Value:1.832%<br>Retention time:39.640 min.
n-Pentadecane	ACAL-APR-37-00	Value:0.119%<br>Retention time:16.249 min.
Phytane	ACAL-APR-37-00	Value:0.921%<br>Retention time:34.307 min.
Squalene	ACAL-APR-37-00	Value:0.376%<br>Retention time:34.913 min.

Approved:

Dr. Nagwa Abo El-Maali

ACAL Lab Manager

ACAL
Analytical Chemistry Unit Assiut University

Analytical Chemistry Unit (ACAL)
www.acal.edu.eg Tel: +20(88)2412315
info@acal.edu.eg Fax: +20 (88) 2080799
Mob: +20(101)9343637
+20(115)2854546
Assiut University
ACAL

Data file: D:\MassHunter\GCMS\1\data\sample 2022\09-18-2022\sample Marjoran 01.D

Method :ACAL-APR-37-00

Customer reference: Marjoran

Sample ID: 9006573

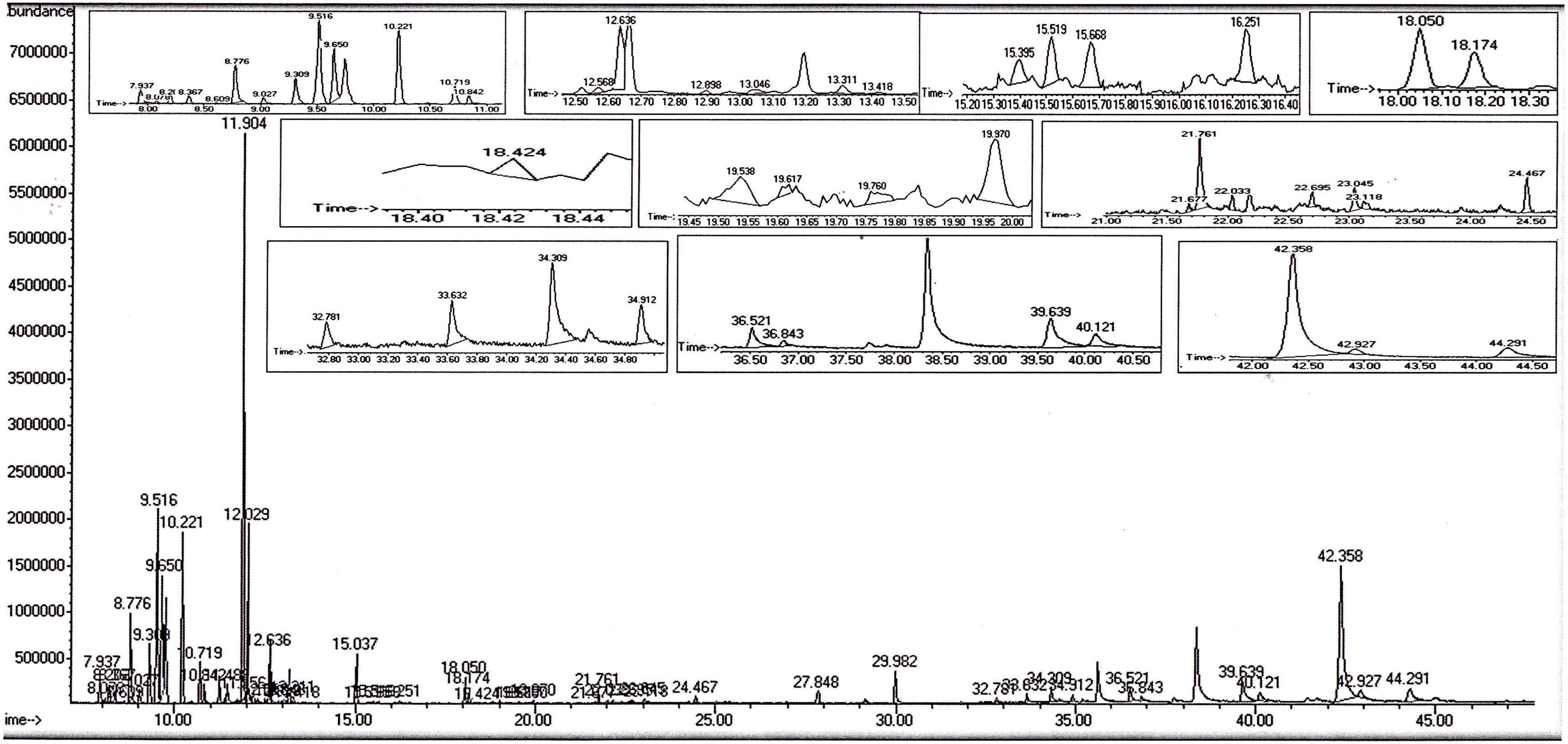

Supplement: Supplementary file 1 [file life-13-00664-s001.zip › life-2186066-supplementary-Report S1.pdf]
